# Identification and genomic characterisation of known and novel highly divergent sapoviruses in frugivorous and insectivorous bats in Nigeria

**DOI:** 10.1080/29986990.2025.2503155

**Published:** 2025-06-11

**Authors:** Uwem E. George, Lander De Coninck, Oluwadamilola A. George, Taiye Adeyanju, Arthur Oragwa, Joshua Kamani, Joseph Igbokwe, Andrew Adamu, Temitope Faleye, Richard Adeleke, Tomiwa Adesoji, Timothy K. Soh, Oluyomi Sowemimo, Philomena Eromon, Olubusuyi M. Adewumi, Johnson A. Adeniji, Onikepe Folarin, Scott C. Weaver, Anise Happi, Jens B. Bosse, Robert W. Cross, Isaac Komolafe, Jelle Matthijnssens, Christian Happi

**Affiliations:** a Institute of Genomics and Global Health (formerly African Centre of Excellence for Genomics of Infectious Diseases), Redeemer’s University, Ede, Nigeria; b Department of Microbiology and Immunology, University of Texas Medical Branch, Galveston, TX, USA; c Department of Microbiology, Immunology and Transplantation, Division of Clinical and Epidemiological Virology, Laboratory of Viral Metagenomics, KU Leuven, Rega Institute, Leuven, Belgium; d Ibadan Diagnostic and Epidemiology Laboratory, National Veterinary Research Institute, Ibadan, Oyo State, Nigeria; e Department of Wildlife and Ecotourism Management, Faculty of Renewable Natural Resources, University of Ibadan, Ibadan, Nigeria; f Department of Veterinary Microbiology, Faculty of Veterinary Medicine, University of Jos, Jos, Nigeria; g Parasitology Division, National Veterinary Research Institute (NVRI), Vom, Nigeria; h Department of Zoology, Faculty of Science, Obafemi Awolowo University, Ile Ife, Nigeria; i Division of Tropical Health and Medicine, Australian Institute of Tropical Health and Medicine, James Cook University, Townsville, Australia; j College of Public Health, Medical and Veterinary Sciences, James Cook University, Douglas, Australia; k Department of Veterinary Public Health and Preventive Medicine, University of Abuja, Abuja, Nigeria; l Center for Environmental Health Engineering, Biodesign Institute, Arizona State University, Tempe, AZ, USA; m Department of Immunology and Infectious Diseases, College of Veterinary Medicine, Cornell University, New York, NY, USA; n Department of Microbiology, Obafemi Awolowo University, Ile-Ife, Nigeria; o Hannover Medical School (MHH), Institute of Virology, Hannover, Germany; p Leibniz Institute of Virology (LIV), Hamburg, Germany; q Centre for Structural Systems Biology (CSSB), Hamburg, Germany; r Cluster of Excellence RESIST (EXC 2155), Hannover Medical School, Hannover, Germany; s Department of Virology, College of Medicine, University of Ibadan, Ibadan, Nigeria; t Infectious Disease Institute, College of Medicine, University of Ibadan, Ibadan, Nigeria; u Department of Biological Sciences, Faculty of Natural Sciences, Redeemer’s University, Ede, Nigeria; v Department of Immunology and Infectious Diseases, Harvard T H Chan School of Public Health, Boston, MA, USA

**Keywords:** Sapovirus, bats, metagenomic sequencing, protein modelling, VP1 protein, new genogroups, Nigeria

## Abstract

Sapovirus (SaV) infections have been linked with moderate-to-severe acute gastroenteritis (AGE) in animals and humans and represent a significant risk to public health. SaVs from animals including pigs, chimpanzees, and rodents have been reported to be closely related with human SaVs, indicating the possibility of cross-species transmission. Divergent SaVs have been reported in various bat species across various continents including Asia, Europe, Oceania and Africa. However, little is known about the evolutionary history of SaVs across various bat species and their zoonotic potential. In this report, we describe the findings of a surveillance study across various bat species in Nigeria. Samples were pooled and subjected to metagenomics sequencing and analyses. Nine of 57 sample pools (containing 223 rectal swabs from five bat species) had SaV reads from which we assembled a total of four complete and three near-complete (having complete coding sequences) genomes. The bat SaV (BtSaV) strains from this study formed five distinct lineages of which four represented novel genogroups. BtSaV lineages clustered mainly according to bat families, which might suggest a likely virus-host-specific evolution. The BtSaV VP1 capsid protein structure prediction confirmed three main domains (S, P1, and P2) as reported for Human SaV (HuSaV). We found that the P2 subdomain of the VP1 protein contains a degree of homology to known immunoreactive epitopes suggesting these conserved regions may be valuable for diagnostics or medical countermeasure development. This study expands our understanding of reservoir hosts, provides information on the genetic diversity and continuous evolution of SaVs in bats.

## Introduction

*Sapovirus* alongside *Norovirus* is one of the 11 genera in the family *Caliciviridae*, having only one species (*Sapporo virus*) [1]. Sapoviruses (SaVs) were initially identified in faecal samples collected from infants as part of a stool survey in Glasgow children using electron microscopy [2]. SaV infections have been associated with moderate-to-severe acute gastroenteritis (AGE) in animals and humans and represent a significant risk to public health [3,4]. Currently, there are no specific therapeutics or vaccines available for the management and prevention of SaV diseases.

The SaV virion consists of a non-enveloped icosahedral capsid (typically 30–38 nm in diameter) containing the positive-sense, single-stranded RNA genome [5]. The RNA genome has two or three open reading frames (ORFs), depending on the SaV genogroup. The large ORF1 polyprotein encodes both non-structural proteins (NS1-7) and the major capsid protein (VP1) while the small ORF 2 encodes VP2 (a minor structural protein) [6]. The VP1 protein of SaV, like that of other caliciviruses, is the main capsid component of the SaV virion, and it is critical for immunological response as well as determining SaV genetic variation and genotypes [7]. The VP1 protein consists of approximately 560 amino acids (aa) organised into two main domains: the protruding (P) domain and the shell (S) domain, plus an N-terminal arm (NTA) which connects the S domain in the capsid shell [8,9]. The P domain is made up of two subdomains: P1 and P2, with P2 being the outermost virion structure. Hypervariable regions on the P2 subdomain have been shown to serve as the primary targets of neutralising antibodies [10].

SaVs are classified into genogroups based on amino acid (aa) sequence identity analysis of the complete VP1 amino acid sequences [11]. Recently, a dual classification criterion has been proposed, with cutoff values for genogroup clusters defined as <0.503 (VP1) and <0.531 (RdRp). Based on these criteria, SaVs have been classified into 34 genogroups (GI to GXXI and GNA1 – GNA3). SaVs can be further classified into genotypes, with cutoff values for genotype clusters for VP1 and RdRp sequence identity defined as <0.161 and <0.266. Currently, at least 52 genotypes have been reported within the genus Sapovirus [12]. SaVs have been reported in a wide range of hosts, including domestic pigs [13], chimpanzees [14], foxes, hyenas, lions [15], rats [16], and humans [17,18].

Human SaVs fall into four genogroups (GI, GII, GIV and GV) and some SaVs detected in animal hosts have been found to cluster closely with human SaVs especially members in genogroups GI, GV, and GII [14,16,19]. While this suggests that SaVs are likely to cross host barriers, it does not indicate the direction of transmission.

Bats are the second largest group of mammals after the order Rodentia, accounting for more than 20% of all known mammalian species worldwide [20]. They are extensively distributed in nature and contribute significantly to both the biological and ecological diversity of numerous habitats. In Africa, anthropogenic factors especially land-use change and overexploitation have resulted in bats being heavily hunted [21]. This has resulted in a rise in the number of bat colonies located close to or inside urban environments, as well as more frequent encounters with humans, pet animals, and livestock, which may increase the risk for zoonotic disease outbreaks. Recently, divergent SaVs have been reported in various bat species across Asia [22], Europe [23], Oceania [24] and Africa [25]. However, little is known about the evolutionary history of SaV across various bat species and their zoonotic potential.

Most of the recent discoveries of SaVs in bats were based on the use of enhanced pathogen discovery techniques including metagenomics. This approach has been pivotal not just in the discovery of distinct strains of SaV in humans and animals but also in our improved understanding of the disease burden of SaVs [17,18,24,25]. In this study, we report the identification and genomic characterisation of highly divergent SaVs in both fructivorous and insectivorous bats in Nigeria using next-generation sequencing. We also provide further insight into the genetic diversity of SaVs across various bat species. Furthermore, using molecular modelling, we predicted the VP1 and VP2 structures of the detected bat SaVs (BtSaV), their unique antigenic sites and B cell epitopes in the immunodominant VP1 domain. Overall, these data are vital for improving SaV classification, viral taxonomy, developing effective diagnostic tests, and gaining a better knowledge of SaV evolution and host species adaptability.

## Materials and methods

### Ethical authorisation and sample collection

**Figure 1.**
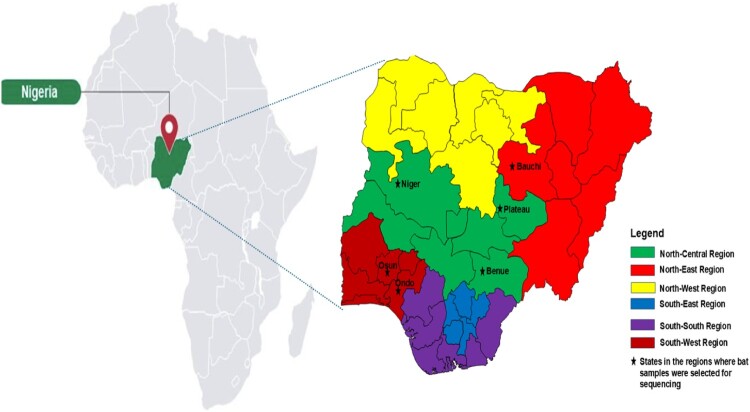
Map of Africa highlighting Nigeria and the various geographical regions where bat samples were selected for sequencing in this study.

The bat rectal swabs analysed in this study were collected as part of the Nigerian bat virome project from insectivorous bats (*Mops condylurus*, *Chaerephon spp*. and *Hipposideros ruber*) and fruit-eating bats (*Rousettus aegyptiacus* and *Eidolon helvum*) from 6 states (Benue, Bauchi, Ondo, Niger, Osun and Plateau) in Nigeria between 2019 and 2022 (Figure 1 and Table S1). Bats were captured around caves and fruit trees at night using harp traps and mist nets as previously described [26]. Morphological features were assessed for each captured bat to determine species, and molecular confirmation of bat species was done as previously described [27] using primers targeting cytochrome b (Cyt b) and mitochondrial cytochrome oxidase subunit 1 (COI). Briefly, rectal swabs were collected and placed in tubes containing 1 mL of virus transport medium. The bat sample collection was done in a microbiological safety station, with personal protective equipment on. The samples were transported in – 20°C containers to the African Centre of Excellence for Genomics of Infectious Diseases (ACEGID) laboratory at Redeemer's University in Nigeria, where they were stored at – 80°C until processed. A total of four hundred and twenty samples were collected (409 between 2019 and 2021 [26] and 11 samples in 2022). The National Veterinary Research Institute (NVRI) Nigeria's Animal Care and Use Committee approved the study design and sampling technique (approval number AEC/03/65/19). We were also given authorisation by the Plateau State Health Research Ethics Committee (approval number PSSH/ADM/ETH.CO/2019/005).

In this study, 223 archived (stored at −80°C) rectal swabs suspended in a virus transport medium were randomly selected and combined into 57 pools based on bat species and the state of collection and subsequently analysed. Approximately 200 µL of the rectal swab suspensions were combined to create each pool, and each contained one to eight faecal suspensions (Table S1).

### Sample preparation and sequencing

Virus enrichment and library preparation were performed using either the unbiased next-generation sequencing technique as previously reported [28] at Institute of Genomics and Global Health (formerly ACEGID), Redeemer’s University, Ede, Nigeria or the NetoVIR protocol [29] at KU Leuven, Rega Institute, Laboratory of Clinical and Epidemiological Virology in Belgium. At KU Leuven, 40 sample pools were analysed using the NetoVIR protocol. Briefly, Fecal suspensions were filtered through a 0.8 μm PES filter and free-floating nucleic acids digested using a combination of Micrococcal Nuclease (New England Biolabs, Ipswich, MA, USA) and Benzonase (Millipore, Billerica, MA, USA). Nucleic acid was then extracted using the QIAamp Viral RNA Mini Kit (Qiagen, Hilden, Germany). For first – and second-strand synthesis, a slightly modified Whole Transcriptome Amplification (WTA2) Kit procedure (Sigma-Aldrich, St Louis, MO, USA) was used, followed by 17 cycles of random PCR amplification. The Nextera XT Library Preparation Kit (Illumina, San Diego, CA, USA) was used to prepare the libraries after which sequencing was performed on the Illumina NextSeq 500 platform (300 cycles, 2 × 150 bp paired ends). At the Institute of Genomics and Global Health formerly ACEGID, 17 individual samples were processed and sequenced. Briefly, after elution, the RNA was treated with turbo DNase to remove any contaminating DNA. The cDNA was then synthesised using a Superscript III Synthesis kit (Invitrogen) and random primers. Sequencing libraries were prepared using the Illumina Nextera XT kit. Subsequently, paired-end sequencing was carried out using the Illumina MiSeq Reagent Kit v2 (500 cycles) on an Illumina Miseq platform (Table S1).

### Read processing, genome assembly and annotation

We processed all raw reads using the Virome Paired-End Reads (ViPER) pipeline (https://github.com/Matthijnssenslab/ViPER), regardless of sequencing platform, to identify reads containing SaVs. In brief, we used Trimmomatic [30] to remove sequencing adapters and improve the quality of the raw reads, and Bowtie2 to eliminate reads that mapped to the host genome [31]. All the trimmed and filtered reads were then de novo assembled into contigs using metaSPAdes [32]. We also performed de novo assembly with MEGAHIT [33] to validate the BtSaV genomes assembled using metaSPAdes. We annotated contigs with DIAMOND (sensitive option) [34] and used BLASTn to further validate the assembled SaV contigs. Using Bowtie2 [31], trimmed reads were mapped against the SaV contigs to assess coverage depth. We identified SaV Open Reading Frames (ORFs) using the ORF finder analysis tool and MetaGeneMark (https://genemark.bme.gatech.edu/genemark/meta_gmhmmp.cgi version 3.25) [35].

### Phylogenetic and recombination analysis

Following a BLASTn search with SaV contigs from this study as queries, we retrieved five sequences from GenBank for each contig with highest identity and coverage. We also retrieved all available SaV reference sequences from the thirty-four documented SaV genogroups from NCBI virus database, along with high-quality BtSaV genomes meeting criteria of <10% ambiguous nucleotides and 80% alignment length. Post-deduplication, we created two sets of data: one comprising the alignment of SaVs found in our research alongside all reference SaV sequences (GI – GXXI and GNA1 – GNA3) from both humans and animals (including complete genome and VP1-only reference sequences for comparison) and another dataset consisting of all BtSaVs found worldwide with a minimum of 80% genome coverage (to assess the genetic diversity of SaVs in different bat species/families). We utilised MAFFTv7.505 [36] to align the data and reconstructed phylogenies using IQTREE2 [37] with ModelFinder [38]. The phylogenetic trees were visualised using Interactive Tree of Life (iTOL) v6 [39]. To further validate the divergence and pairwise identity of our BtSaV sequences and published reference sequences, we aligned each distinct pair using the Sequence Demarcation Tool [40]. We examined all the BtSaV sequences for recombination using RDP5 [41].

### Molecular modelling and structural prediction of bat Sapovirus VP1 proteins

We aligned the VP1 domain of Nigerian BtSaV with known VP1 sequences from human SaVs (AB455803.1 and AJ606694.2) to identify conserved regions and amino acid substitutions related to potential binding to human receptors. The S, P1, and P2 domains in the VP1 protein were the focus of the analysis. Physical and general biological properties of the bat SaV VP1 proteins were calculated using ProtParam and ProtScale tools on the ExPASy Server (accessible at https://web.expasy.org/cgi-bin/protparam/protparam). To predict regions in the BtSaV VP1 protein sequences that are likely to be antigenic (antigenic epitopes), a previously described approach [42] with an antigen prediction tool (accessible at http://imed.med.ucm.es/Tools/antigenic.pl) was used.

To predict potential B-cell epitopes present within the BtSaV VP1 domain, we employed the Bepipred Linear Epitope Prediction 3.0 tool [43]. Protein structure prediction was performed with LocalColabFold v1.5.2 [44] (https://github.com/YoshitakaMo/localcolabfold) [45] to generate 5 models using 20 recycles and a stop-at-score of 100. AlphaFold 3 [46] was used through the AlphaFold Server (https://alphafoldserver.com). Visualisation was performed with ChimeraX version 1.8 (https://www.cgl.ucsf.edu/chimerax) [47].

## Results

### Bat sample demographic, Sapovirus diversity and phylogenetic analysis

We screened a total of 223 rectal swabs from 5 bat species (*Mops condylurus*, *Eidolon helvum*, *Chaerephon spp*, *Hipposideros ruber* and *Rousettus aegyptiacus*) collected from six Nigerian states (Benue, Bauchi, Ondo, Niger, Osun and Plateau) (Figure 1), which were divided over 57 pools (**Table 1** **and** Table S1).

**Table 1.** Demographic description and summary of 9 pools containing Sapovirus reads.

S/N	Pool ID	Year of sample collection	Location	Bat family	Bat species	Total reads	SaV Mapped reads	Number of Sapovirus contigs
1.	B8/BT/OAU/2	2020	Osun	*Pteropodidae*	*E. helvum*	12,731,940	155,425	2
2.	B12/BT/OAU/6	2020	Osun	*Pteropodidae*	*E. helvum*	14,935,074	248	2
3.	B14/BT/OAU/8	2020	Osun	*Pteropodidae*	*E. helvum*	15,225,166	27,224	5
4.	B15/BT/OAU/9	2020	Osun	*Pteropodidae*	*E. helvum*	1,447,204	46	1
5.	B17/BT/OAU/11	2020	Osun	*Pteropodidae*	*E. helvum*	10,948,508	429	1
6.	B23/BT/OAU/17	2020	Osun	*Pteropodidae*	*E. helvum*	3,975,982	153	3
7.	GB09	2020	Benue	*Molossidae*	*Mops condylurus*	381,938	10,853	2
8.	G10	2022	Ondo	*Pteropodidae*	R. aegyptiacus	4,001,878	22,270	1
9.	G11	2022	Ondo	*Pteropodidae*	R. aegyptiacus	3,314,134	586	1

Of the 57 pools, we detected SaVs reads in 9 pools (15.8%). Out of the 9 pools with SaV reads, 6 pools were derived from straw-coloured fruit bat (*Eidolon helvum*) (Osun State), 2 from *Rousettus aegyptiacus* (Ondo State), and 1 from *Mops condylurus* (Benue State) (Table 1). No SaV reads were detected in samples from the Bauchi, Niger and Plateau states. We assembled a total of 4 complete, 3 near-complete (having complete coding sequences) and 11 partial genomes ranging in length from 7611–7898 (Table S2**)**.

**Figure 2.**
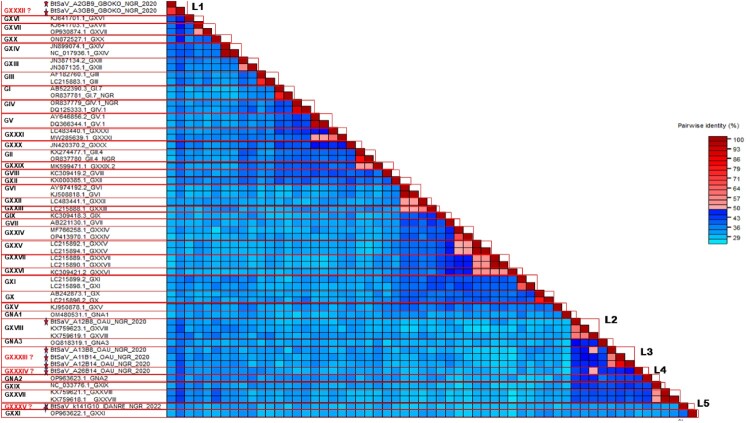
Classification of SaV (GI to GXXI and GNA1 – GNA3) based on cutoff values for genogroup clusters defined as <0.503 and cutoff values for genotype clusters defined as <0.161 using SDT showing evidence of several tentative new genogroups. SaV contigs detected in this study are highlighted in red font with a question mark and a star while previously described SaVs are in black font.

To determine the divergence of the BtSaV strains detected in this study, BtSaV sequences (we focused mostly on the four complete, three near-complete and one partial genome having over 70% genome coverage with complete VP1 and VP2) and all previously described reference SaV genogroup sequences (GI to GXXI and GNA1 – GNA3) were subjected to Sequence Demarcation tool analysis. The analysis was based on cutoff values for genogroup clusters defined as <0.503 and cutoff values for genotype clusters defined as <0.161) [12]. We found that the Nigerian BtSaV sequences formed 5 distinct lineages (L1 – L5) and only one of the BtSaV from this study (BtSaV/A12B8/OAU/NGR/2020 – L2) belonged to a previously described genogroup (GXXII), which consisted of BtSaV detected in Eidolon helvum bats from Cameroon. The remaining BtSaV from this study were likely members of four novel genogroups, tentatively named GXXXII to GXXXV (Figure 2).

**Figure 3.**
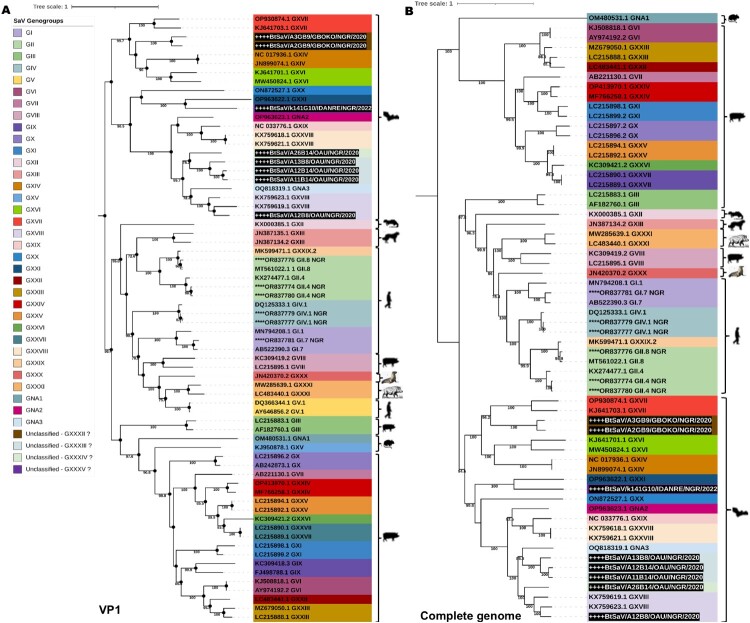
Maximum likelihood trees of SaV GI – GXXI and GNA1 – GNA3 based on **A.** complete VP1 gene and **B.** Complete genome sequences. All SaV genogroups were assigned a specific colour according to the legend provided. The BtSaV strains reported in this research are marked with an asterisk and highlighted in white font and black background while previously reported HuSaV from Nigeria are highlighted in black asterisk.

To further investigate the genetic diversity of the Nigerian BtSaV lineages in relation to SaV from humans and other animals, we generated two phylogenetic trees using available dataset of SaV reference sequences (GI to GXXI and GNA1 – GNA3). The first tree was based on complete VP1 regions, since it is the most representative region and often used for typing of SaV and the second tree was based on complete genomes sequences (where available). In our phylogenetic analyses, we found that the BtSaV strains from this study formed 5 distinct lineages (like the SDT analysis) with high support (bootstrap = 100) in both phylogenies, suggesting the co-circulation of divergent BtSaVs in Nigerian bats (Figure 3A, B). The BtSaVs from this study were all distantly related to human SaVs (HuSaVs) including the recently reported HuSaVs from Nigeria (Figure 3A) [17].

**Figure 4.**
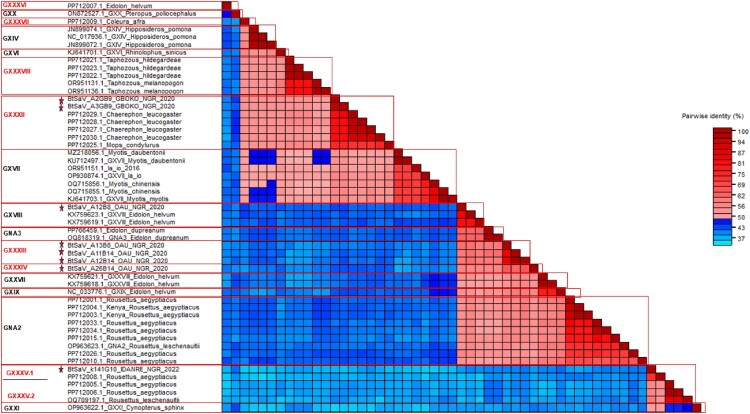
Classification of BtSaV based on cutoff values for genogroup clusters defined as <0.503 and cutoff values for genotype clusters defined as <0.161 using SDT showing evidence of 17 distinct BtSaV lineages detected in various bat species. Tentative new genogroups are highlighted in red font with a question mark and red star while previously described BtSaVs are in black font.

**Figure 5.**
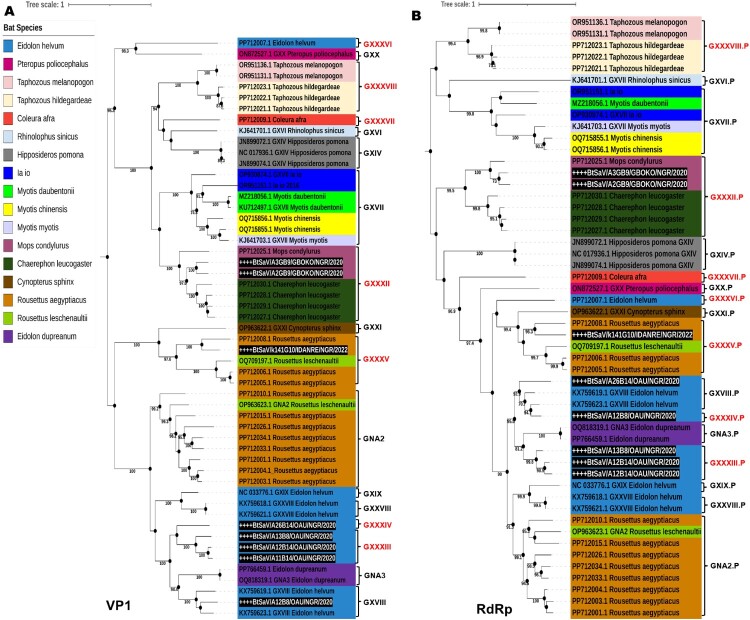
Maximum likelihood tree of All BtSaV based on **A.** complete VP1 gene and **B.** Complete RdRp gene, with 1000 bootstrap replications. Bat species are assigned a specific colour according to the legend provided. The BtSaV strains reported in this research are marked with an asterisk and highlighted in white.

Next, we focused our attention specifically to bat SaVs. We constructed a phylogenetic tree of the complete VP1 and RdRp gene to assess the genetic diversity of SaVs in different bat species/families using BtSaV sequences from this study and BtSaVs found worldwide (sequences with a minimum of 80% genome coverage). Our maximum likelihood (ML) tree results showed that a total of 17 bat species in six different bat families were found to be infected with SaVs. Based on SaV genogroup demarcation criteria using SDT, we found that at least 17 distinct SaV lineages may be circulating in various bat species globally with species members in the *Pteropodidae* family harbouring the highest number of divergent SaVs (Figures 4 and 5 and Figure S1). We also found that various BtSaV lineages clustered mainly according to bat families, which suggests virus-host-specific evolution. Furthermore, we observed that species within some bat families (especially species members in the families *Molossidae* and *Vespertilionidae*), harboured genetically related BtSaV irrespective of location where bats were sampled. For instance, SaVs detected in *Mops condylurus* bats (species member in *Molossidae* family) in this study from Nigeria were genetically related to SaVs detected in *Chaerephon leucogaster* bats sampled in Kenya (Figure S1).

Based on boot scan analysis in RDP5, the BtSaV sequences found in this study did not contain any evidence for recombination events.

## Physicochemical characterisation and B-cell epitope prediction of bat sapovirus VP1 protein

To determine the physicochemical characteristics of the BtSaV VP1 protein detected in this study, the sequences were submitted to the ProtParam database for analysis. We found that the BtSaV VP1 ranged from 534 to 544 amino acids while their molecular weights were between 55.8 and 57.6 kDa (Table S3).

To determine epitopes in the VP1 domain that may serve as targets for immune recognition, the BtSaV sequences along with a reference human SaV (HuSaV) sequence (AB455803) were subjected to the Predicting Antigenic Peptide software. Overall, we found that the BtSaV had antigenic determinants across the VP1 domain ranging from 23 - 27 epitopes (Figure S2).

**Figure 6.**
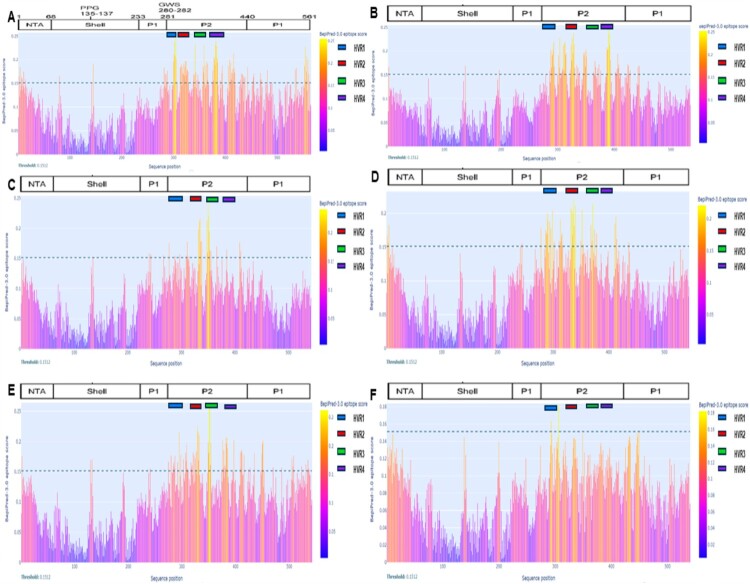
Predicted target sites for B cell binding across the VP1 domain with threshold set at 0.15. (A). Human SaV AB455803, (B) BtSaV/A2GB9/GBOKO/NGR/2020, (C) BtSaV/A11B14/OAU/NGR/2020, (D) BtSaV/A13B8/OAU/NGR/2020, (E) BtSaV/A26B14/OAU/NGR/2020 and (F) BtSaV/k141G10/IDANRE/NGR/2022. The hypervariable regions (HVR) located within the P2 subdomain in the VP1 are colour-coded as shown in the legend.

Based on the Bepipred Linear Epitope Prediction analysis of potential B-cell epitopes in the BtSaV VP1 domain, we observed a preponderance of B-cell epitopes located in the P2 subdomain; mostly in the hypervariable regions (HVR) 1 - 4 of both HuSaVs and BtSaVs (Figure 6). Determining the specific regions of highly probable B cell epitopes, is of great practical interest and could be crucial for the design and development of potent and highly effective anti-SaV vaccines.

## The virion architecture is conserved within the *Caliciviridae* family

Our analysis to gain insight into the conserved regions and amino acid substitutions related to potential binding to human receptors in the S, P1, and P2 domains in the VP1 protein of the BtSaV in comparison with HuSaV showed minor conserved motifs in the S and P1 domains. The P2 domain alignment (containing the hypervariable regions), known to be the target of neutralising antibodies [10], differed greatly within both HuSaVs and BtSaVs (Figure S3). To examine the mean distance between the Cα atoms of HuSaV and BtSaV and ascertain the structural similarity of immunogenic and antigenic regions in HuSaV VP1 protein in relation to BtSaVs, the predicted VP1 protein structure of BtSaV/A2GB9/GBOKO/NGR/2020 was compared to that of HuSaV (pdb: 7dod). It was discovered that the P1 and a section of the P2 subdomain (crucial for antigen binding) showed moderate-to-high conservation (Figure S5).

**Figure 7.**
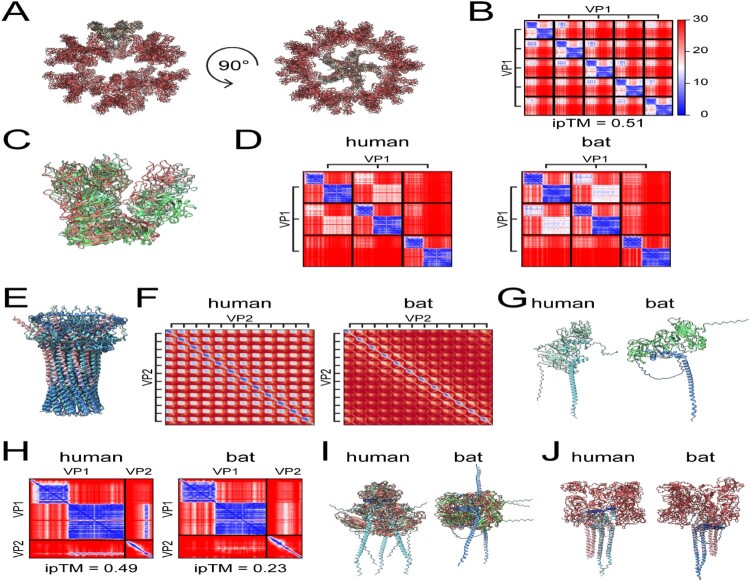
Structure predictions of VP1 and VP2 propose conserved virion architecture and an important α-helix in VP2. (A) LocalColabFold prediction of a pentamer of VP1 from HuSaV AJ606694.2 (light green) reproduces the 5-fold axis from the HuSaV VLP structure^4^ (red). (B) The PAE plot of the pentamer structure in (A) shows protein-protein interactions. This heat map scheme is used for all subsequent PAE plots. (C) LocalColabFold predictions of a trimer of VP1 from HuSaV AJ606694.2 (light green) and BtSaV/A2GB9/GBOKO/NGR/2020 (green) reproduce the asymmetric unit from the HuSaV VLP structure^4^ (red). (D) The PAE plots of the trimers in (C) show protein-protein interactions. (E) AlphaFold 3 predictions of a dodecamer of VP2 from HuSaV AJ606694.2 (light blue) or BtSaV/A2GB9/GBOKO/NGR/2020 (blue) reproduces the VP2 portal barrel of cat FCV^5^ (pink). (F) The PAE plot of the dodecamers in (E) shows protein-protein interactions. (G) LocalColabFold prediction of the VP1-VP2 heterodimer (green and blue, respectively) from HuSaV AJ606694.2 and BtSaV/A2GB9/GBOKO/NGR/2020. (H) The PAE plots of the VP1-VP2 heterodimers in (G) predict an interaction of VP1 with an α-helix in VP2. This heterodimer was aligned to all 3 conformations of VP1 in the HuSaV VLP^9^ (red) (I) or both conformations of VP2 in cat FCV^48^ (pink) (J). The interacting α-helix in VP2 is shown in dark blue.

To further characterise the structural proteins, structure predictions were generated of oligomers of VP1 and/or VP2 from HuSaV AJ606694.2 and BtSaV/A2GB9/GBOKO/NGR/2020. The VP1 pentamer aligns to the 5-fold axis of the solved structure of a HuSaV VLP [9] (Figure 7, A-B), the VP1 trimer aligns to the asymmetric unit in the T = 3 VLP [9] (Figure 7, C-D), and the VP2 dodecamer aligns to the receptor-induced portal dodecamer from feline calicivirus (FCV) [48] (Figure 7, E-F). While the human virus generates a high confidence dodecamer, the bat virus prediction is less confident but still exhibits the same organisation. These recapitulated higher-order macromolecular structures suggest a conserved virion structure between caliciviruses.

Structure predictions of VP1-VP2 implicates a conserved α-helix in human and bat VP2 in stabilising the heterodimer (Figure 7, G-H). The prediction of the human virus has higher confidence than the bat virus. Since the bat virus still shows the same pattern, collectively it suggests a common feature. Aligning the heterodimer into each of the VP1 positions in the VLP asymmetric unit [48] displays clashes with VP2 (Figure 7, I). This suggests that VP2 does not fit into the icosahedral packing of VP1 and perhaps incorporation of VP2 disrupts the organisation. This could explain why VP2 is a minor capsid protein and that receptor-induced conformation changes are not coordinated [48]. In contrast, aligning the heterodimer into the portal complex [48] points this α-helix towards VP1 (Figure 7, J). Collectively, this suggests that a C-terminal α-helix in VP2 interacts with VP1 without complete occupancy in the virion.

## Discussion

We report the detection of 4 complete, 3 near-complete (having complete coding sequences) and 11 partial genomes of SaVs from rectal swabs collected from frugivorous and insectivorous bats in Nigeria using metagenomics. This is the first report of SaV in Nigerian bats, and our analysis revealed the co-circulation of highly divergent novel SaV lineages in Nigerian bats. Specifically, we document the presence of five distinct lineages, representing probably 4 novel genogroups with one lineage belonging to a previously reported genogroup (GXVIII) (Figures 2 and 3). SaV infections in both humans and animals have been associated with cases of AGE, posing a significant public health issue [4]. SaVs from animals including pigs, chimpanzees, and rodents have been reported to be closely related with human SaVs, indicating the possibility of cross-species transmission [14,16]. There is a need for continuous surveillance to generate full-length or partial genome sequences of SaVs to guide the development of therapeutics and diagnostic tests, enhance viral taxonomy and classification, and provide insight into the molecular mechanisms influencing the host adaptation and evolution of SaVs in humans and animals.

While PCR surveillance of SaV has shown success in identifying circulating genotypes in humans and animals, this method may not be effective in detecting novel or highly divergent lineages of SaV, resulting in partial knowledge of SaV circulation. The utilisation of primer-independent deep sequencing has led to the identification of divergent SaVs in both human and animal populations [17,22–25].

We also found that a high proportion (66%) of the distinct SaV lineages were in straw-coloured fruit bat species (Table 1, Figures 3 and 5). A similar preponderance of distinct BtSaV in straw-coloured fruit bat species was reported in Cameroon, which suggests that this bat species may play a vital role as a reservoir in the circulation and evolution of SaV [25]. The straw-coloured fruit bat is the most widespread and commonly hunted bat species in Africa and often roosts in urban areas, increasing its opportunities to encounter humans [21,49]. Therefore, it is crucial to comprehend the mechanisms of SaV persistence and transmission dynamics in this species to avoid future spillover.

The BtSaV identified in this study were not closely related to HuSaVs, including the SaVs recently reported in children from Nigeria [17] (Figure 3). However, in our global assessment of the genetic diversity of SaVs in different bat species/families, we discovered that distinct BtSaV lineages grouped primarily according to bat families (Figure 5 and Figure S1) with species members within the bat family harbouring genetically related viruses regardless of bat sampling location or country. Kemenesi et al. [30] reported a similar geographic distant evolutionary relationship between similar BtSaV lineage from European and Asian bats. This could reflect virus-host-specific evolution due to a predilection of certain SaV lineages for bat species in the respective families.

Our analysis of potential B-cell epitopes in the BtSaV VP1 domain showed that most B-cell epitopes are in the P2 subdomain, specifically in the hypervariable regions (HVR) 1–4 (Figure 6). The main goal of predicting B cell epitopes in protein sequences is to identify specific segments that can be used to generate specific antibodies instead of using the whole protein. Linear B cell epitope prediction is also essential for developing synthetic peptides to generate antibodies targeting specific antigens. Our comparison of the BtSaV VP1 structure to HuSaV revealed three main domains (S, P1, and P2), similar to the reported structure of mature HuSaV capsid protein [9]. The moderate to high conservation in the P1 and a section of the P2 subdomain in comparison with HuSaV (crucial domain for antigen binding and primary targets of neutralising antibodies) [10] (Figure 6) suggests potential similarities in receptor motifs and antigenic determinants. The protein folding in the P1 and P2 subdomains is noted to be conserved across caliciviruses [7]. However, a key distinction in the sequence of BtSaVs and HuSaV was the presence of various minor insertions and deletions in the P2 subdomain (Figure S4). Comparable changes in the P2 region have been noted in both HuSaVs and BtSaV [9,30] and could result in longer loops on the exteriors. Nevertheless, experimental research is required to comprehend the interactions between these novel BtSaVs and receptors on the surface of the bat host cells and to clarify the impact of this interaction on viral pathogenesis and regulation of morbidity and mortality in the bat host.

Some of our study's limitations included nucleic acid degradation caused by previous freeze–thaw procedures, which may have influenced the quantity and quality of genomes recovered in the present investigation. We were also unable to assess the true prevalence of SaV in individual samples because of the small sample volume of the archived samples.

In conclusion, we report the detection and genomic characterisation of novel SaVs from frugivorous and insectivorous bats in Nigeria. This study extends our knowledge about the genetic diversity and geographical distribution of SaVs as well as the diversity of host species and ongoing evolution of SaV in bats. Our findings showed that the VP1 protein, specifically the P2 subdomain, contains immunoreactive epitopes which can serve as targets for development of an anti-SaV vaccine and antiviral medications for treating SaV infections.

## Author contributions

UEG, RWC, IK, JM and CH designed the study. UEG, OG, TA (Adeyanju), AO, JK, JI, AA (Adamu), RA, TA (Tomiwa) and OS collected the samples; UEG, LDC, PE and JM performed the molecular assays and metagenomic sequencing; UEG, LDC, TF, TKS and JM conducted the bioinformatics analysis and protein modelling; UEG and TKS wrote the initial draft manuscript; SCW, AH, OF, JM, OMA, JAA, JBB, RWC, IK and CH reviewed and edited the manuscript; JBB, RWC, IK, JM and CH supervised the work; OMA, JAA, JBB, SCW, RWC, IK, JM and CH provided mentorship. UEG, SCW, JBB, RWC, JM and CH facilitated funding acquisition. All the authors read and approved the final manuscript before submission.

## Supplementary Material

Supplementary_files (2).docx

## Data Availability

The bat sapovirus sequenced raw reads for this study and the generated genomes have been deposited in NCBI under BioProject PRJNA1185806 with accession numbers PQ623340-PQ623357.
