# Supplementary material for: Identification and genomic characterisation of known and novel highly divergent sapoviruses in frugivorous and insectivorous bats in Nigeria
Source: EMI Anim Environ. 2025 Jun 11;1(1):2503155. doi: 10.1080/29986990.2025.2503155 (PMC12080456; doi:10.1080/29986990.2025.2503155)
Supplement: Supplementary_files (2).docx [file TEMA_A_2503155_SM4149.docx]

**Title: Identification and Genomic Characterization of Known and Novel Highly Divergent Sapoviruses in Frugivorous and Insectivorous Bats in Nigeria**

**Table S1: Demographic description and summary of bat samples analysed in this study**

| **S/N** | **Sample ID** | **Site of sample collection** | **Bat Family** | **Bat Species** | **Year** | **Individual or pool** |
| --- | --- | --- | --- | --- | --- | --- |
| 1 | BA1 | Gboko-Benue state | *Molossidae* | *Mops Condylurus* | 2019 | Pool of 5 bats |
| 2 | BA2 | Gboko-Benue state | *Molossidae* | *Mops Condylurus* | 2019 | Pool of 5 bats |
| 3 | BA3 | Mkar-Benue state | *Pteropodidae* | *Eidolon helvum* | 2019 | Pool of 5 bats |
| 4 | BA4 | Lim- Bauchi state | *Pteropodidae* | *Eidolon helvum* | 2019 | Pool of 5 bats |
| 5 | BA5**^a^** | Lim, Bauchi state | *Pteropodidae* | *Eidolon helvum* | 2019 | Pool of 5 bats |
| 6 | BA6 | Vom-Jos, Plateau state | *Molossidae* | Chaerephon spp | 2019 | Individual |
| 7 | B7/BT/OAU/1 | OAU-Osun state | *Pteropodidae* | *Eidolon helvum* | 2020 | Pool of 5 bats |
| 8 | B8/BT/OAU/2 **^b, c^** | OAU-Osun state | *Pteropodidae* | *Eidolon helvum* | 2020 | Pool of 5 bats |
| 9 | B9/BT/OAU/3 | OAU-Osun state | *Pteropodidae* | *Eidolon helvum* | 2020 | Pool of 5 bats |
| 10 | B10/BT/OAU/4 | OAU-Osun state | *Pteropodidae* | *Eidolon helvum* | 2020 | Pool of 5 bats |
| 11 | B11/BT/OAU/5 | OAU-Osun state | *Pteropodidae* | *Eidolon helvum* | 2020 | Pool of 5 bats |
| 12 | B12/BT/OAU/6 **^b, c^** | OAU-Osun state | *Pteropodidae* | *Eidolon helvum* | 2020 | Pool of 5 bats |
| 13 | B13/BT/OAU/7 | OAU-Osun state | *Pteropodidae* | *Eidolon helvum* | 2020 | Pool of 5 bats |
| 14 | B14/BT/OAU/8 **^b, c^** | OAU-Osun state | *Pteropodidae* | *Eidolon helvum* | 2020 | Pool of 5 bats |
| 15 | B15/BT/OAU/9 **^b, c^** | OAU-Osun state | *Pteropodidae* | *Eidolon helvum* | 2020 | Pool of 6 bats |
| 16 | B16/BT/OAU/10 | OAU-Osun state | *Pteropodidae* | *Eidolon helvum* | 2020 | Pool of 4 bats |
| 17 | B17/BT/OAU/11 **^b, c^** | OAU-Osun state | *Pteropodidae* | *Eidolon helvum* | 2020 | Pool of 4 bats |
| 18 | B18/BT/OAU/12 | OAU-Osun state | *Hipposideridae* | *Hipposideros ruber* | 2020 | Pool of 5 bats |
| 19 | B19/BT/OAU/13 | OAU-Osun state | *Hipposideridae* | *Hipposideros ruber* | 2020 | Pool of 6 bats |
| 20 | B20/BT/OAU/14 | OAU-Osun state | *Hipposideridae* | *Hipposideros ruber* | 2020 | Pool of 5 bats |
| 21 | B21/BT/OAU/15 | OAU-Osun state | *Hipposideridae* | *Hipposideros ruber* | 2020 | Pool of 5 bats |
| 22 | B22/BT/OAU/16 | OAU-Osun state | *Pteropodidae* | *Eidolon helvum* | 2020 | Pool of 4 bats |
| 23 | B23/BT/OAU/17 **^b, c^** | OAU-Osun state | *Pteropodidae* | *Eidolon helvum* | 2020 | Pool of 2 bats |
| 24 | B24/BT/PL/01 | Jos Zoo, Plateau state | *Pteropodidae* | *Eidolon helvum* | 2021 | Pool of 3 bats |
| 25 | B25/BT/PL/02 | Jos Zoo, Plateau state | *Pteropodidae* | *Eidolon helvum* | 2021 | Pool of 3 bats |
| 26 | B26/BT/PL/03 | Jos Zoo, Plateau state | *Pteropodidae* | *Eidolon helvum* | 2021 | Pool of 3 bats |
| 27 | B27/BT/PL/04 | Jos Zoo, Plateau state | *Pteropodidae* | *Eidolon helvum* | 2021 | Pool of 8 bats |
| 28 | B28/BT/PL/05 | Jos Zoo, Plateau state | *Pteropodidae* | *Eidolon helvum* | 2021 | Pool of 8 bats |
| 29 | B29/BT/PL/06 | Jos Zoo, Plateau state | *Pteropodidae* | *Eidolon helvum* | 2021 | Pool of 8 bats |
| 30 | B30/BT/PL/07 | Jos Zoo, Plateau state | *Pteropodidae* | *Eidolon helvum* | 2021 | Pool of 8 bats |
| 31 | B31/BT/PL/08 | Jos Zoo, Plateau state | *Pteropodidae* | *Eidolon helvum* | 2021 | Pool of 8 bats |
| 32 | B32/BT/PL/09 | Jos Zoo, Plateau state | *Pteropodidae* | *Eidolon helvum* | 2021 | Pool of 7 bats |
| 33 | B33/BT/PL/010 | Jos Zoo, Plateau state | *Pteropodidae* | *Eidolon helvum* | 2021 | Pool of 7 bats |
| 34 | B34/BT/GB/01 | Gboko-Benue state | *Molossidae* | *Mops Condylurus* | 2020 | Pool of 5 bats |
| 35 | B35/BT/GB/02 | Gboko-Benue state | *Molossidae* | *Mops Condylurus* | 2020 | Pool of 5 bats |
| 36 | B36/BT/GB/03 | Gboko-Benue state | *Molossidae* | *Mops Condylurus* | 2020 | Pool of 5 bats |
| 37 | B37/BT/BA/01 | Lim, Bauchi state | *Pteropodidae* | *Eidolon helvum* | 2019 | Pool of 5 bats |
| 38 | B38/BT/BA/02 | Lim, Bauchi state | *Pteropodidae* | *Eidolon helvum* | 2019 | Pool of 5 bats |
| 39 | B39/BT/BA/03 | Lim, Bauchi state | *Pteropodidae* | *Eidolon helvum* | 2019 | Pool of 5 bats |
| 40 | B40/BT/BA/04 | Lim, Bauchi state | *Pteropodidae* | *Eidolon helvum* | 2019 | Pool of 5 bats |
| 41 | GB04 | Gboko-Benue state | *Molossidae* | *Mops Condylurus* | 2020 | Individual |
| 42 | GB09 **^b, d^** | Gboko-Benue state | *Molossidae* | *Mops Condylurus* | 2020 | Individual |
| 43 | GB10 | Gboko-Benue state | *Molossidae* | *Mops Condylurus* | 2020 | Individual |
| 44 | GB12 | Gboko-Benue state | *Molossidae* | *Mops Condylurus* | 2020 | Individual |
| 45 | GB13 | Gboko-Benue state | *Molossidae* | *Mops Condylurus* | 2020 | Individual |
| 46 | NG17 | Paiko - Niger state | *Molossidae* | *Chaerephon spp* | 2021 | Individual |
| 47 | NG19 | Paiko - Niger state | *Molossidae* | *Chaerephon spp* | 2021 | Individual |
| 48 | NG22 | Paiko - Niger state | *Molossidae* | *Chaerephon spp* | 2021 | Individual |
| 49 | NG24 | Paiko - Niger state | *Molossidae* | *Chaerephon spp* | 2021 | Individual |
| 50 | NG33 | Paiko - Niger state | *Molossidae* | *Chaerephon spp* | 2021 | Individual |
| 51 | NG34 | Paiko - Niger state | *Molossidae* | *Chaerephon spp* | 2021 | Individual |
| 52 | NG35 | Paiko - Niger state | *Molossidae* | *Chaerephon spp* | 2021 | Individual |
| 53 | CER24 | OAU-Osun state | *Hipposideridae* | *Hipposideros ruber* | 2021 | Individual |
| 54 | CER43 | OAU-Osun state | *Hipposideridae* | *Hipposideros ruber* | 2021 | Individual |
| 55 | PL66 | Jos Zoo, Plateau state | *Pteropodidae* | *Eidolon helvum* | 2021 | Individual |
| 56 | G10 **^b, d^** | Idanre – Ondo state | *Pteropodidae* | *R. aegyptiacus* | 2022 | Individual |
| 57 | G11 **^b, d^** | Idanre – Ondo state | *Pteropodidae* | *R. aegyptiacus* | 2022 | Individual |

Abbreviations: Obafemi Awolowo University, OAU;

**^a^** This sample was excluded from sequencing after library preparation due to low DNA concentration and contamination.

**^b^** Samples with Sapovirus reads detected.

**^c^** Samples where Sapovirus reads were detected using the NetoVIR protocol [37].

**^d^** Samples where Sapovirus reads were detected using the Matranga et al. [36] protocol.

**Table S2: Summary of SaV contigs detected in the pooled bat samples**

| **S/N** | **Virus strain/Isolate name** | **Accession number** | **Nucleotide length** | **Mean Coverage** | **Genome completeness** | **SaV Genogroup** |
| --- | --- | --- | --- | --- | --- | --- |
| 1. | BtSaV/A2GB9/GBOKO/NGR/2020 | PQ623354 | 7587 | 214.3 | Complete genome | Unclassified |
| 2. | BtSaV/A3GB9/GBOKO/NGR/2020 | PQ623355 | 7533 | 101 | Complete genome | Unclassified |
| 3. | BtSaV/A12B8/OAU/NGR/2020 | PQ623340 | 7611 | 2,912 | Near-complete genome with complete coding sequence | GXVIII |
| 4. | BtSaV/A13B8/OAU/NGR/2020 | PQ623341 | 7453 | 220 | Near-complete genome with complete coding sequence | Unclassified |
| 5. | BtSaV/A11B14/OAU/NGR/2020 | PQ623344 | 7483 | 521 | Near-complete genome with complete coding sequence | Unclassified |
| 6. | BtSaV/A12B14/OAU/NGR/2020 | PQ623345 | 7361 | 372 | Near-complete genome with complete coding sequence | Unclassified |
| 7. | BtSaV/k141G10/IDANRE/NGR/2022 | PQ623356 | 7110 | 467 | Complete genome | Unclassified |
| 8. | BtSaV/A26B14/OAU/NGR/2020 | PQ623346 | 5573 | 32 | Partial genome (complete VP1 and VP2) | Unclassified |
| 9. | BtSaV/A128B12/OAU/NGR/2020 | PQ623342 | 1176 | 9.147 | Partial genome | GXVIII |
| 10. | BtSaV/A141B12/OAU/NGR/2020 | PQ623343 | 1715 | 15 | Partial genome | GXVIII |
| 11. | BtSaV/A97B14/OAU/NGR/2020 | PQ623347 | 2735 | 521.4044 | Partial genome | Unclassified |
| 12. | BtSaV/A347B14/OAU/NGR/2020 | PQ623348 | 1673 | 15 | Partial genome | Unclassified |
| 13. | BtSaV/A93B15/OAU/NGR/2020 | PQ623349 | 898 | 6.8296 | Partial genome | Unclassified |
| 14. | BtSaV/A43B17/OAU/NGR/2020 | PQ623350 | 2293 | 26.2957 | Partial genome | GXVIII |
| 15. | BtSaV/A277B23/OAU/NGR/2020 | PQ623351 | 1883 | 11.3011 | Partial genome | GXVIII |
| 16. | BtSaV/A583B23/OAU/NGR/2020 | PQ623353 | 1288 | 10 | Partial genome | GXVIII |
| 17. | BtSaV/A904B23/OAU/NGR/2020 | PQ623352 | 1036 | 11 | Partial genome | GXVIII |
| 18. | BtSaV/A25G11/IDANRE/NGR/2022 | PQ623357 | 2103 | 40.3428 | Partial genome | Unclassified |


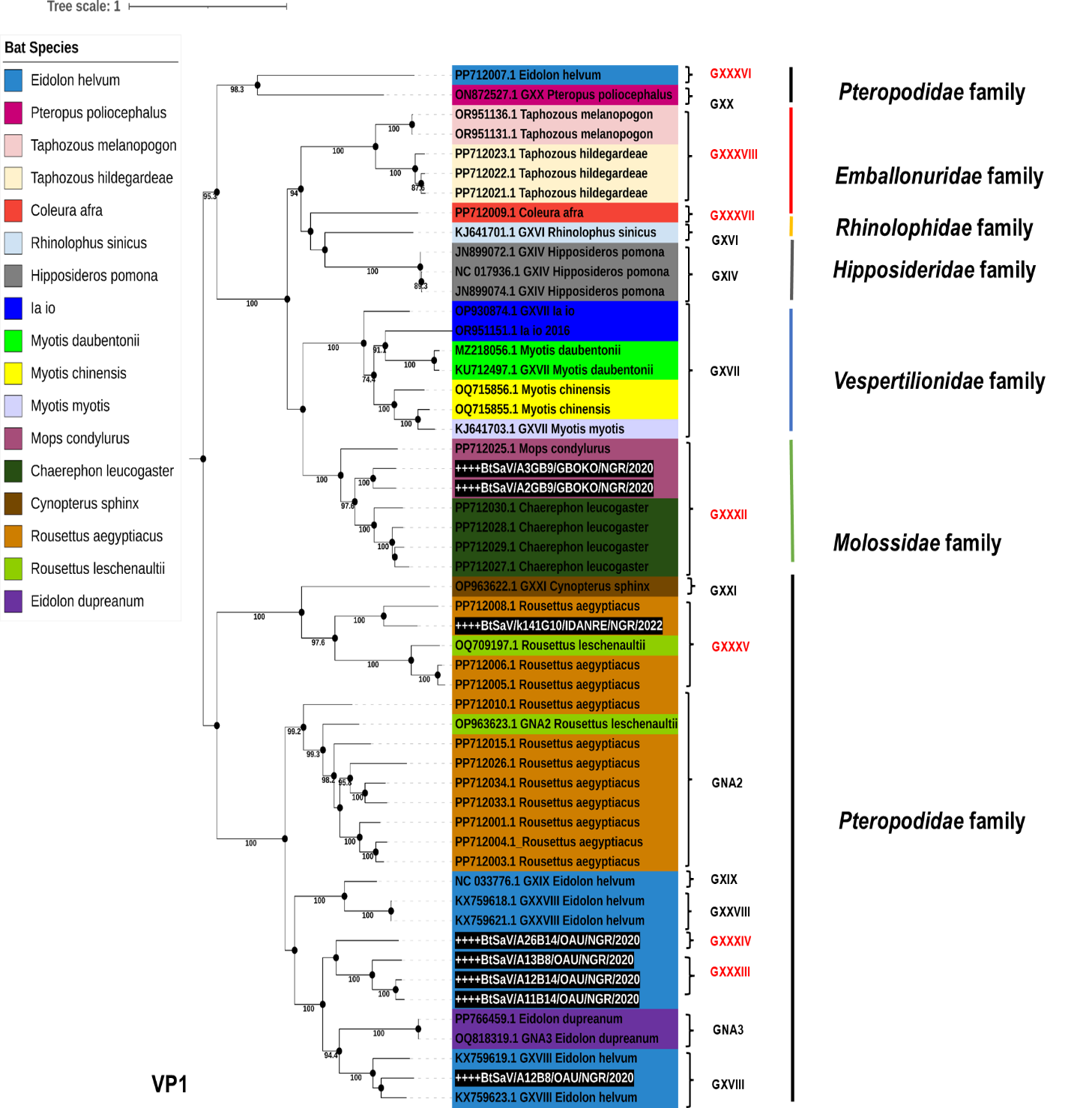


**Figure S1.** Maximum likelihood tree using ModelFinder of All BtSaV based on A. complete VP1 gene, with 1000 bootstrap replications. Bat species and families are assigned a specific colour according to the legend provided. The BtSaV strains reported in this research are marked with an asterisk and labelled in white. Tentative newly described genogroups are highlighted in red. The tree was visualized using the Interactive Tree of Life (iTOL) v6 with midpoint rooting.

**Table S3: Physical and Chemical properties of BtSaV VP1 protein detected in this study.**

| **Characteristic** | **BtSaV/A2GB9/GBOKO/NGR/2020** | **BtSaV/A3GB9/GBOKO/NGR/2020** | **BtSaV/A12B8/OAU/NGR/2020** | **BtSaV/A13B8/OAU/NGR/2020** | **BtSaV/A11B14/OAU/NGR/2020** | **BtSaV/A12B14/OAU/NGR/2020** | **BtSaV/k141G10/IDANRE/NGR/2022** | **BtSaV/A26B14/OAU/NGR/2020** |
| --- | --- | --- | --- | --- | --- | --- | --- | --- |
| Number of amino acids | 534 | 536 | 544 | 544 | 542 | 541 | 540 | 542 |
| Formula | C_2508_H_3843_N_677_O_747_S_24_ | C_2514_H_3855_N_677_O_754_S_24_ | C_2542_H_3937_N_687_O_777_S_17_ | C_2543_H_3969_N_705_O_787_S_17_ | C_2527_H_3961_N_693_O_781_S_19_ | C_2527_H_3960_N_677_O_747_S_19_ | C_2472_H_3879_N_659_O_780_S_15_ | C_2534_H_3976_N_694_O_786_S_13_ |
| Molecular weight | 56200.63 | 56396.79 | 57099.38 | 57555.76 | 57155.56 | 57120.56 | 55791.84 | 57156.40 |
| Theoretical isoelectric point (PI) | 5.23 | 5.23 | 5.37 | 5.41 | 5.40 | 5.40 | 5.30 | 5.55 |
| Number of negatively charged residues | 40 | 40 | 37 | 35 | 33 | 33 | 32 | 34 |
| Number of positively charged residues | 25 | 25 | 28 | 25 | 23 | 23 | 23 | 25 |
| Instability index (II) | 26.84 (stable) | 28.37 (stable) | 35.56 (stable) | 29.79 (stable) | 32.51 (stable) | 30.31  (stable) | 27.70  (stable) | 29.26  (stable) |
| Aliphatic index | 78.88 | 78.92 | 90.04 | 85.09 | 87.97 | 88.30 | 86.98 | 88.49 |
| Grand average of hydropathicity (GRAVY) | 0.010 | 0.004 | 0.021 | -0.025 | 0.047 | 0.040 | 0.155 | 0.045 |


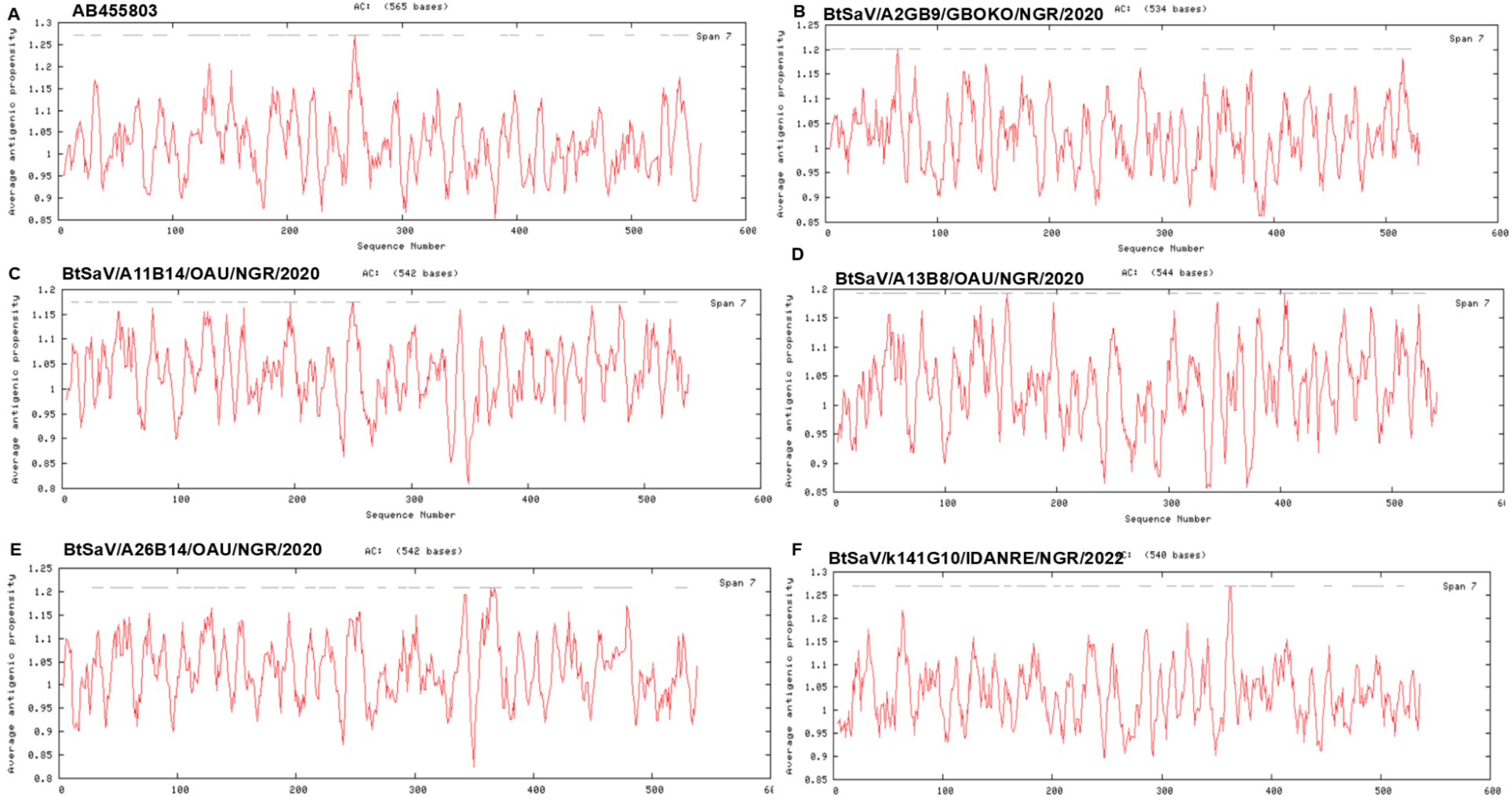


**Figure S2.** Analysis of antigenic determinants in the VP1 protein. (A). AB455803 VP1 protein showing 24 epitopes, (B). BtSaV/A2GB9/GBOKO/NGR/2020 VP1 protein showing 24 epitopes, (C). BtSaV/A11B14/OAU/NGR/2020 VP1 protein showing 27 epitopes, and (D). BtSaV/A13B8/OAU/NGR/2020 VP1 protein showing 25 epitopes, (E). BtSaV/A26B14/OAU/NGR/2020 VP1 protein showing 23 epitopes and (F). BtSaV/k141G10/IDANRE/NGR/2022 VP1 protein showing 23 epitopes respectively.


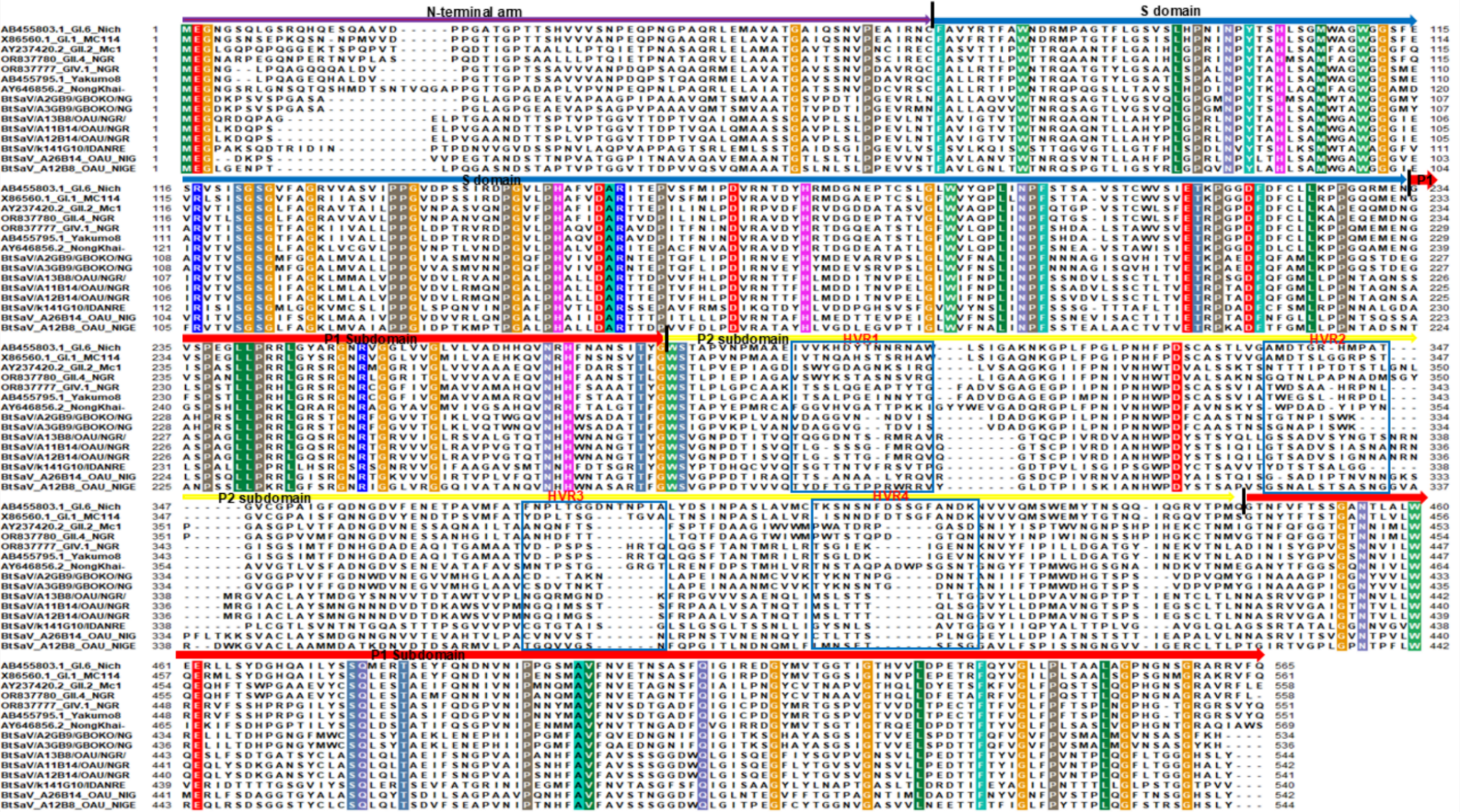


**Figure S3**: Sequence alignment of HuSaV and BtSaV complete VP1 protein. conserved motifs are coloured across the residue. The N-terminal arm is highlighted in purple, the S-domain in blue, the P1 subdomain in red and the P2 subdomain in yellow. Hypervariable regions (HVR) 1 - 4 are also highlighted in blue.


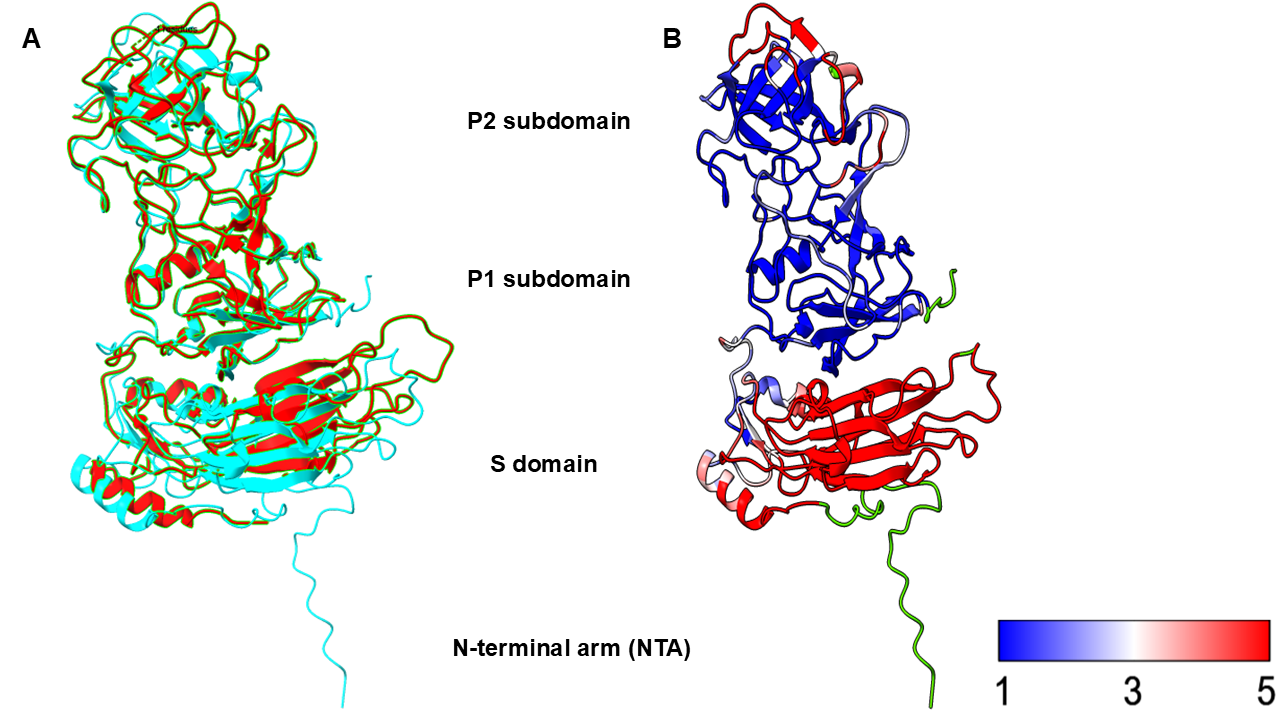


**Figure S4.** Comparing the similarity and average distance between the Cα atoms of superimposed HuSaV (pdb: 7dod) and BtSaV/A2GB9/GBOKO/NGR/2020. (**A**) The HuSaV VP1 protein template structure is shown in red cartoon, while BtSaV/A2GB9/GBOKO/NGR/2020 VP1 model structure is depicted in cyan cartoon. (**B**) Conservation of amino acid residues of BtSaV/A2GB9/GBOKO/NGR/2020 VP1 proteins mapped onto the molecular surface of HuSaV template to show the average distance between their Cα atoms. Blue indicates that the root mean square deviation (RMSD) of Cα atoms are within 1.0-Å between the two proteins while Red indicates that the RMSD of Cα atoms of the two proteins are 5.0-Å or more away from each other. The portion-coloured green indicates the region with no correspondence between the two proteins.
